# A Simple Histone Code Opens Many Paths to Epigenetics

**DOI:** 10.1371/journal.pcbi.1002643

**Published:** 2012-08-16

**Authors:** Kim Sneppen, Ian B. Dodd

**Affiliations:** 1 Niels Bohr Institute/CMOL, University of Copenhagen, Copenhagen, Denmark; 2 Molecular and Biomedical Science (Biochemistry), University of Adelaide, Adelaide, Australia; MRC Laboratory of Molecular Biology, United Kingdom

## Abstract

Nucleosomes can be covalently modified by addition of various chemical groups on several of their exposed histone amino acids. These modifications are added and removed by enzymes (writers) and can be recognized by nucleosome-binding proteins (readers). Linking a reader domain and a writer domain that recognize and create the same modification state should allow nucleosomes in a particular modification state to recruit enzymes that create that modification state on nearby nucleosomes. This positive feedback has the potential to provide the alternative stable and heritable states required for epigenetic memory. However, analysis of simple histone codes involving interconversions between only two or three types of modified nucleosomes has revealed only a few circuit designs that allow heritable bistability. Here we show by computer simulations that a histone code involving alternative modifications at two histone positions, producing four modification states, combined with reader-writer proteins able to distinguish these states, allows for hundreds of different circuits capable of heritable bistability. These expanded possibilities result from multiple ways of generating two-step cooperativity in the positive feedback - through alternative pathways and an additional, novel cooperativity motif. Our analysis reveals other properties of such epigenetic circuits. They are most robust when the dominant nucleosome types are different at both modification positions and are not the type inserted after DNA replication. The dominant nucleosome types often recruit enzymes that create their own type or destroy the opposing type, but never catalyze their own destruction. The circuits appear to be evolutionary accessible; most circuits can be changed stepwise into almost any other circuit without losing heritable bistability. Thus, our analysis indicates that systems that utilize an expanded histone code have huge potential for generating stable and heritable nucleosome modification states and identifies the critical features of such systems.

## Author Summary

Specialized enzymes add and remove chemical modifications to the histone proteins that package DNA into nucleosomes. These modifications act as labels to recruit various proteins to the DNA locations where they are needed to control DNA functions, such as gene expression. The modifications are usually made and maintained in response to specific signals. However, if a modifying enzyme is itself recruited by the modification it makes, then this positive feedback could cause the modification or its absence to be self-sustaining, and even heritable, once the signal has gone. We used computer simulations to systematically explore the possibilities for such epigenetic states when there is an expanded modification ‘code’ - one that involves the presence or absence of two different modifications rather than just one. We found that this small expansion of the histone code allows hundreds of different modification and enzyme recruitment schemes to give alternative stable and heritable states. These worked best when the nucleosomes in alternative states were differently modified at both positions. All working schemes involved positive feedback and cooperativity between nucleosomes. Thus, even a simple histone code could be used in many ways to make stable and heritable, yet reversible, marks on DNA.

## Introduction

The histone proteins that form the nucleosomes that package eukaryotic DNA are subject to various post-translational modifications of several of their exposed amino acid residues. These chemical modifications are added and removed by a large number of specific enzymes, and create the potential for a vast number of different nucleosome types. Specific modifications (i.e. particular chemical modifications of particular histone residues) affect the binding of other proteins to nucleosomes, which in turn can affect the packaging, replication, recombination, repair and expression of the underlying DNA. Many different protein domains or modules have been shown to confer modification- sensitive nucleosome binding [1]. The presence of multiple modification ‘reader’ domains within a single protein or protein complex inspired the histone code hypothesis - that specific combinations of different modifications can have distinct downstream consequences [2]. More recent experimental observations support this idea. First, high resolution ChIP analysis has shown that complex patterns of histone modifications are associated with specific sequences [3]. Although ChIP does not prove co-existence of modifications on single nucleosomes, top-down mass spectrometry has revealed over 100 different modification patterns on individual histone proteins in vivo [4], [5]. Secondly, there is now a number of examples, at least in vitro, of multivalent reader proteins distinguishing different combinations of modifications at multiple histone positions [6]–[9].

Combining a protein element that can recognize a specific nucleosome modification with a protein element that can catalyze (‘write’) the same modification creates the possibility for positive feedback and bistability [10]–[14]. The idea is that specifically modified nucleosomes recruit enzymes that cause other nearby nucleosomes to become similarly modified. This feedback and the distribution of parental nucleosomes to nearby locations on both daughter DNA molecules after DNA replication [15], means that alternative modification states could be persistent and heritable within a cluster of nucleosomes. Such states are believed to provide for epigenetic regulation of the underlying genes, allowing transient signals to produce the long-term and heritable expression states needed for cell differentiation and development. Indeed, several histone modifying enzymes involved in epigenetic regulation are known to recognize and create the same modification [16]–[19].

**Figure 1:**
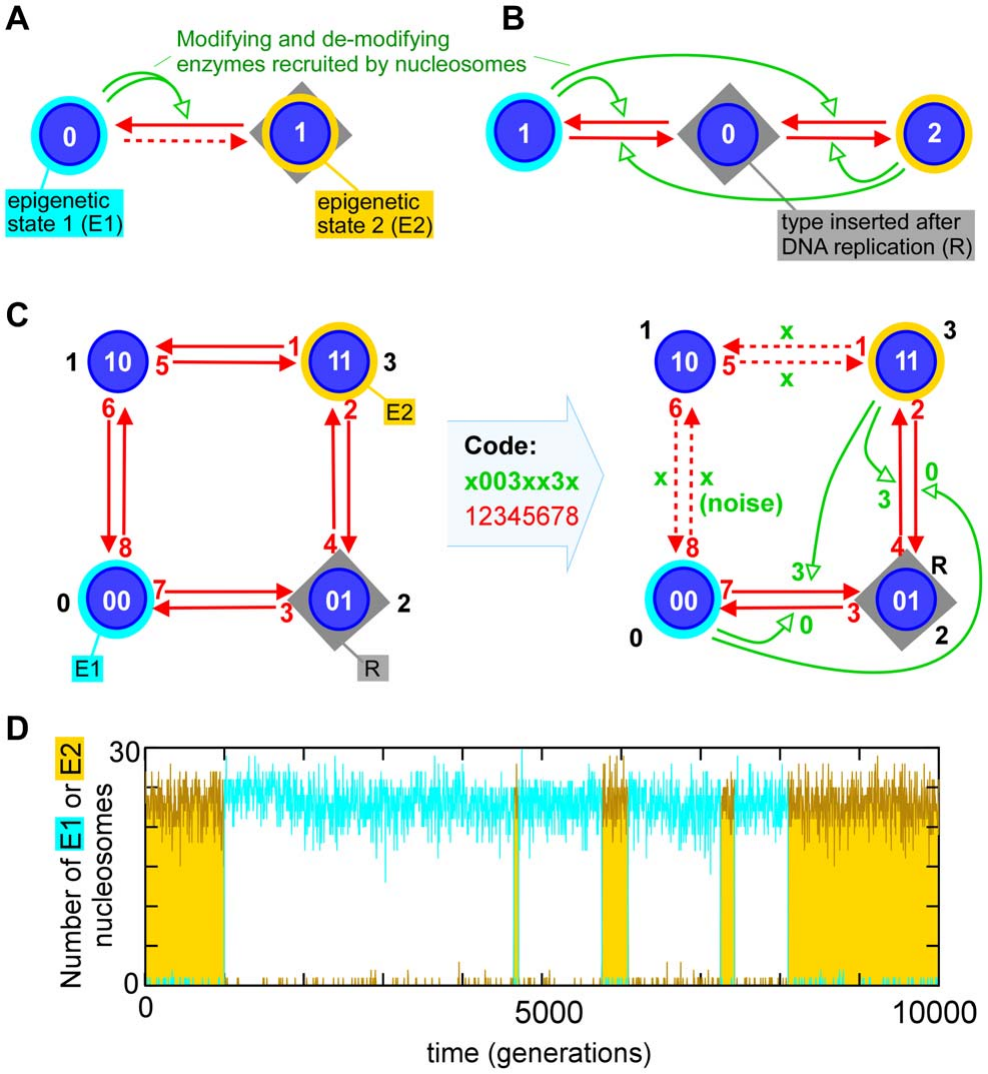
Model for nucleosome modification circuits, using a code with two histone modifications. (A) A simple modification scheme previously shown to be capable of giving heritable bistability [24], [25] in which one residue per nucleosome is either unmodified (0) or modified (1), generating two nucleosome types, each associated with a distinct epigenetic regulatory state (E1 or E2). The grey box indicates the nucleosome type (R) that is added after DNA replication, which causes roughly half of the nucleosomes in the system to be converted to the R type. Interconversion reactions (red arrows) may occur at a rate that is unaffected by nearby nucleosomes (dashed red arrows) or are stimulated by enzymes recruited by other nucleosomes (green arrows). The green double-tailed arrow indicates direct cooperativity due to recruitment by two nucleosomes. (B) A three nucleosome-type scheme also shown to give heritable bistability [11], [21], in which a single residue per nucleosome can exist in three modification states (e.g. H3K9ac, H3K9 and H3K9me). (C) Extended scheme analyzed here in which each nucleosome can be modified (1) or not (0) at two different histone positions, making a total of 4 nucleosome types (00, 10, 01, 11; e.g. H3K4K9, H3K4meK9, H3K4K9me, H3K4meK9me). [Note that our model effectively only considers the modification combinations for each half-nucleosome (one copy of each histone protein), whereas two binary modifications on each half nucleosome give 10 different full-nucleosome types (00/00, 00/01, 00/10, 00/11, 01/01, 01/10, 01/11, 10/10, 10/11, 11/11)]. Enzymatic transitions between types are by addition or removal of one modification. Each of the 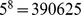 patterns of recruitment-reaction connections are defined by a specific circuit code that lists the recruiting nucleosome (0, 1, 2, 3) for each of the eight reactions; non-recruited ‘noise’ transitions are denoted ‘x’. We term the specific circuit shown in (C) the “classical” motif due to its similarity with the previously studied three nucleosome-type motif shown in (B). (D) Time course displaying strong bistability for the circuit in (C), showing the numbers of E1 and E2 nucleosomes in a 30 nucleosome system (with noise level 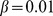).

Theoretical analyses of such systems [11], [20]–[25] have shown that in order to generate heritable bistability, the positive feedback recruitment reaction must be: (1) non-local on the DNA (i.e. involving interactions between non-adjacent nucleosomes), (2) substantially more frequent than non-recruited changes in modification state (such as by histone replacements, random modifications and DNA replication), and (3) cooperative. We have previously examined two ways in which the cooperativity requirement can be met. Direct or explicit cooperativity involves the modifying enzyme needing at least two nucleosomes of a particular type in order to create a new nucleosome of that type (Fig. 1A), for example, if the enzyme is only efficiently recruited by simultaneous contact with two or more nucleosomes [24], [25]. Indirect, or two-step, cooperativity occurs when creation of a particular nucleosome type requires two successive modification steps, each of which can be catalysed by an enzyme recruited by just one nucleosome of that type [11], [21]. Two-step cooperativity requires at least three nucleosome types, which can be achieved using a single histone residue if there are at least three modification states of that residue e.g. a particular lysine may be unmodified, acetylated or methylated (Fig. 1B).

Modeling has so far been confined to systems involving recognition and modification reactions at single histone residues (e.g. Fig. 1AB). Here, we analyze the possibilities for generating heritably bistable systems with a minimal histone code in which there are modifications at two separate histone positions.

## Methods

In its simplest form, where each of the two histone positions is allowed only two possible states, unmodified or modified, this system produces four nucleosome types (0, 1, 2 and 3; Fig. 1C). Applying the histone code concept, the enzymes that bind to and modify these nucleosomes should be capable of distinguishing the four combinations. Thus, there are eight specific modification or de-modification reactions capable of causing inter-conversions between the four types. (It should be noted that because each nucleosome has two copies of each histone and has two-fold symmetry, two modification states at each of two histone positions in fact results in 10 distinct nucleosome types - see Fig. 1 legend. Our simplification is that all enzymes in the system are recognizing modifications on one half of a nucleosome - essentially that each nucleosome in our system is comprised of two independent half-nucleosomes).

Each of the four nucleosome types may or may not recruit an enzyme that catalyzes a particular reaction. Thus there are 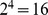 possible combinations for each of the eight reactions, giving a total state space of 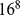 circuits. We reduced this state space by allowing each of the eight reactions to be catalyzed by only one or none of the nucleosome types, giving five possibilities for each reaction and thus 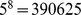 possible circuits. Effectively, we are examining those cases that have maximal discrimination between nucleosome types. One particular circuit (the ‘classical’ circuit, most similar to the 3-nucleosome type system) is shown in Fig. 1C. Each possible circuit is defined by a code assigning, for each reaction, the nucleosome that recruits the enzyme for that reaction. All reactions can also occur by ‘noise’, random transitions occurring irrespective of the status of other nucleosomes in the system. Reactions not subject to recruited modification only occur through this process. We also simplified the system by not adding cooperativity to the individual recruitment reactions and by making all recruitment reactions of equal strength and all noise reactions of equal strength.

In addition, the four nucleosome states should be distinguishable by the reader proteins that control the expression of the underlying genes. A prevalence of a specific nucleosome type (E1) would result in one epigenetic state (e.g. gene activity) while another type (E2) would be associated with the alternative epigenetic state (e.g. gene inactivity). With a four-nucleosome code these E1 and E2 nucleosomes could either be opposite to each other in the circuit (i.e. different at both histone positions) or adjacent to each other (i.e. different at only one histone position).

The final description needed for the circuit is to define the type of nucleosome that is inserted after replication, R (Fig. 1). The R type can be one of the E1 or E2 types (R-in circuit) or one of the other nucleosome types (R-out circuit), giving 4 different circuit types: E-opposite/R-out, E-opposite/R-in, E-adjacent/R-out and E-adjacent/R-in.

The ability of any specific circuit to generate stable and heritable alternative modification states was tested by iteration of recruitment reactions, non-recruitment (noise) reactions and DNA replication steps among a system of 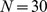 nucleosomes (strictly, 30 half-nucleosomes) as follows.

1. *Recruitment:* A nucleosome 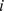 and nucleosome 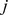 are selected randomly. If 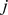 is a type that can catalyze modification of nucleosome 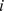 (determined by the circuit), then 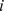 is changed accordingly (if 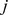 can modify 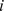 in two ways, one is randomly chosen). If 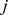 is in no such state, then nothing happens. Note that recruitment is not subject to distance constraints in this scheme; any nucleosome in the system is equally likely to modify any other by recruitment.
2. *Noise:* With probability 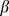, a random nucleosome is changed to one of its neighbor types.
3. *Replication:* At DNA replication, all nucleosomes have a probability 0.5 to be replaced by a nucleosome of the R type. The replication step happens after 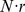 iterations of steps 1 and 2 (one generation). We used 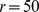, thus for our standard case with 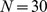, there are about 50 recruitment attempts and 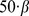 random conversions per nucleosome in the system per generation.

Given a particular circuit we simulate a 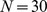 system for 250 generations, starting with all nucleosomes of the E1 type. Subsequently we re-initiate the system by making all nucleosome the alternative E2 type and continue the simulation for another 250 generations. To minimize the effect of randomness of individual simulations, we repeat this procedure 4 times, thus simulating each circuit for a total of 2000 generations.

The simulations are done at low noise, 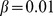, Just before each replication we record the state of the system by counting the number of E1 and E2 nucleosomes, 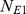 and 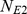. An example of such a time-series is shown in Fig. 1D. The quality of our circuit is evaluated from this time-series.

The first measure, testing for a balanced bistability is
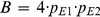
(1)where 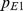 and 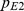 are the respective probabilities for the system as a whole being in one of the epigenetic states. We characterize the system as being in state 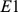 if the number of nucleosome of this type 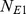 exceeds the alternate type by at least half of the nucleosomes in the system, i.e. 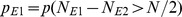 and reversely for 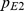.

A 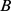 value of 1 corresponds to 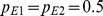. We consider a 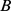 value of 0.75 a threshold for a good balance of the two states; if either 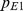 or 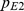 is <0.25 or >0.75, then 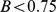. This also allows the system some ‘undecided’ time, since 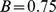 for a system that is in state 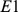 43.3% of the time and in state 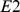 43.3% of the time.

The second score for bistability measures the stability of the epigenetic states, and is simply the frequency of switches between the states per generation 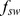. Strongly bistable systems which remain in E1 for the first 250 generations of the simulation and in E2 for the second 250 generations, and so forth for all 4 simulations of each code are assigned a stability 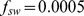.

The third measure of bistability was the average number of generations the system spends in intermediate states when switching between the E1 and E2 states, 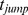. We set a threshold value of 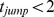, reflecting the decisiveness expected to be important in epigenetic regulatory systems.

In general these measures of bistability were strongly affected by noise, and thus the larger noise a code can sustain, the more robust (to noise) is its associated bistability. Initial screening of circuits was done with a noise level 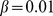.

## Results

### Modifications at two positions allow many bistable circuits

**Figure 2:**
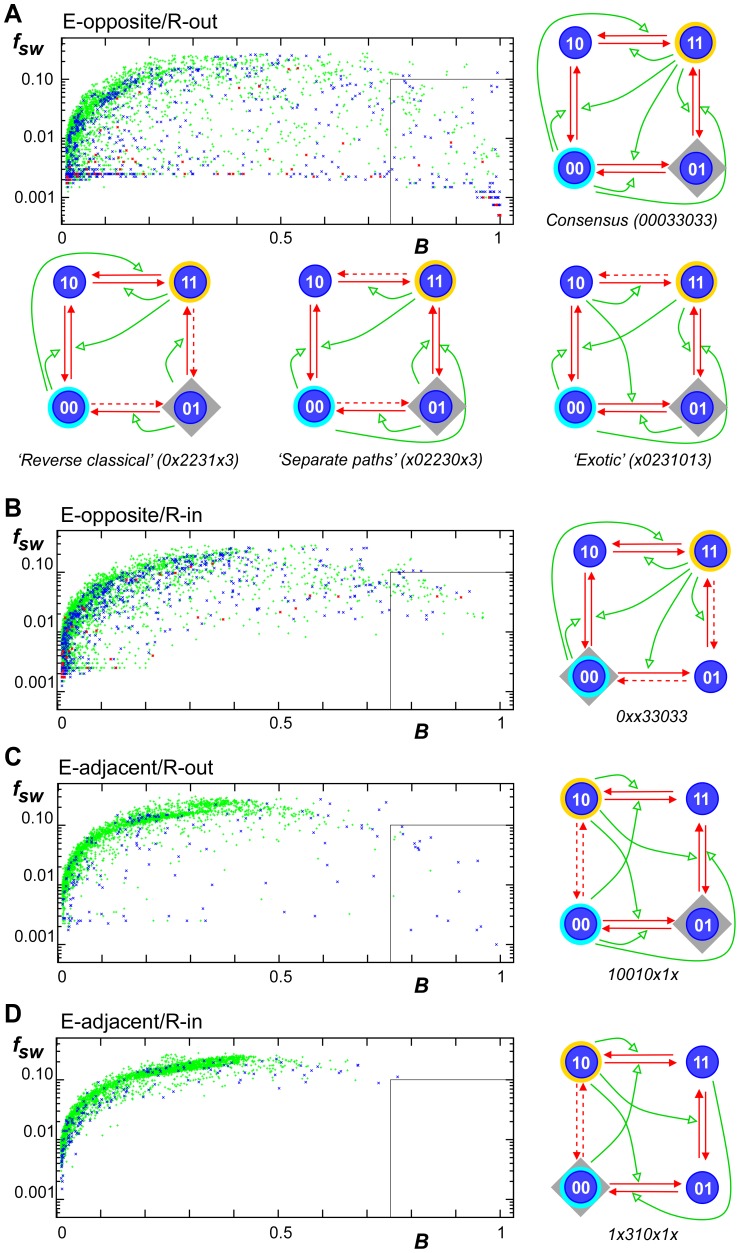
Testing two-modification histone codes for bistability. Simulations were carried out for each of the 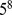 circuits for each of the four different arrangements of the E1, E2 and R nucleosome types (A–D), for a system of 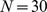 nucleosomes with a noise level 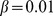. The scatter plots show scores for balance (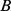) and switching frequency per generation (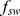) for those circuits that switch reasonably quickly (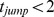)between E1-dominated and E2-dominated states (see Methods). Circuits in the boxed region show high stability (switching less than once per 10 generations) and a reasonable frequency for both the E1 and E2 epigenetic states (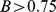). The color of the points indicates the number of two-step positive feedback reactions, where E1 or E2 recruit enzymes that create their own type by two successive reactions. For example, in the consensus motif in (A), 00 nucleosomes cooperate to create their own type by stimulating the 11 to 10 reaction and the 10 to 00 reaction. For green points there are 0 or 1 of these two-step reactions in the motif, for blue there are 2 and for red there are 3 or 4. Selected circuits are shown for each arrangement (symbols as in Fig. 1).

Fig. 2 examines all 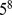 possible circuits for each of the four possible assignments of the E1, E2 and R nucleosome types. The scatter plots show 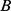 and 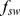 scores for all circuits with 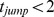. Circuits falling in the lower right side of the plots are the most bistable; we defined ‘working’ circuits as those with 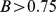, 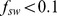 (and 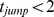). Some of the bistable circuits are shown in Fig. 2. Although we used a low noise level for testing, many of the circuits remained bistable with noise increased four-fold.

We were surprised by the number and variety of circuits able to produce good heritable bistability, especially considering that our search was limited in several ways: 1) It disregarded the possibility that a given enzymatic reaction can be catalyzed by more than one of the 4 nucleosome types. 2) It disregarded that enzymatic reactions and noise moves may be individually graded, and thereby fine tuned to balance an otherwise biased drift among the states. 3) It disregarded the possibility for explicit cooperativity. In addition, our criteria for deciding whether the system was in the E1 or E2 state and for defining reasonable balance between these states were quite stringent.

The number of working circuits can be taken as a measure of mutational robustness for the corresponding E1, E2 and R arrangements. The E-opposite/R-out arrangement was the most robust with 202 working circuits. However the E-opposite/R-in and E-adjacent/R-out arrangements have 67 and 19 working circuits, respectively. The E-adjacent/R-in arrangement gave no bistable circuits by our criteria. These data allow us to conclude:

1. Bistability is most robustly obtained when the alternate epigenetic states are different at both histone positions. This separation allows most easily for two-step cooperativity, where E1 or E2 nucleosomes create themselves by two successive recruitment reactions, one at each nucleosome position. Circuits with at least two of these two-step positive feedback pathways predominate among the working circuits (blue and red dots, Fig. 2). The E-adjacent arrangement tends to short-circuit these two-step pathways. However, in the most bistable E-adjacent circuit (Fig. 2C) the absence of recruited reactions between E1 and E2 allows E1 and E2 nucleosomes to be created predominately through 3-step positive feedback recruitment pathways.
2. Bistability is most robustly obtained if the nucleosome type inserted after replication (R) is not one of the nucleosome types associated with the epigenetic states (E1 or E2). The R-out arrangement produces 3-fold more working circuits than the R-in arrangement. This reflects the difficulty in achieving reasonable balance between the E1 and E2 states when one of them is favored by replication. This imbalance can sometimes be redressed by an opposing imbalance in the number of recruitment reactions favoring the non-R epigenetic state, for example, in the E-opposite/R-in circuit shown in Fig. 2C. We expect that this preference for the R-out arrangement would be relaxed if the relative strengths of recruitment and noise reactions were allowed to vary.

In the following analysis we confine ourselves to the E-opposite/R-out arrangement.

### Modification reactions used by the circuits

**Figure 3:**
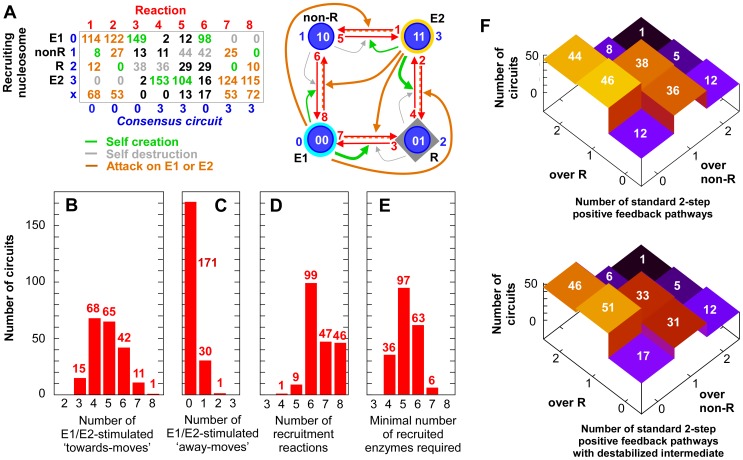
Analysis of the 202 bistable E-opposite/R-out circuits. These circuits have 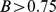, 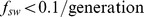 and 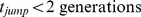. (A) The table shows total numbers of each reaction among the 202 circuits, with types of reactions color-coded. These data are summarized in the circuit, where reaction frequency is denoted by arrow thickness. (B) Number of circuits with the indicated number of recruitment links in which E1 or E2 stimulates moves towards themselves (reactions 1, 2, 3, 6 by E1; 4, 5, 7, 8 by E2). (C) Number of circuits with the indicated number of recruitment links in which E1 or E2 stimulates moves away from themselves (reactions 4, 5, 7, 8 by E1; 1, 2, 3, 6 by E2). (D) Number of recruitment reactions per circuit. (E) Minimal number of recruited enzymes needed for the circuit. This is less than the number of recruitment reactions because in some cases the recruited enzyme acts to modify or de-modify a particular histone position without being sensitive to the modification state of the other position. For example, the consensus motif (Fig. 2A) has 8 recruitment reactions but only requires 4 enzymes. (F) Upper panel: Numbers of circuits with 0, 1 or 2 standard two-step positive feedback pathways stimulated by either E1 or E2 according to whether the pathway is over the R-state, or the non-R state. For example, the ‘classical’ circuit and the ‘reverse classical’ circuit are in the 44 and 12 member classes, respectively. Lower panel: Includes only standard 2-step pathways in which a recruited enzyme catalyzes conversion of the intermediate type to the opposing E type. Most standard two-step pathways are of this type.

The 202 working E-opposite/R-out circuits are examined in more detail in Fig. 3. The number of times that each type of reaction occurs in this group of circuits is shown in Fig. 3A. The most frequent reactions are self-creation recruitment reactions by E1 or E2, particularly from the R state. These reactions provide direct positive feedback by E1 and E2. E1 and E2 also frequently ‘attack’ the opposite state, which not only weakens that state but is the first move towards self-creation. Together, these common reactions generate a consensus circuit in which E1 and E2 each use both possible two-step positive feedback pathways (Fig. 2A). The consensus circuit is the most bistable of the two-modification circuits. In fact, as already seen in Fig. 1D, the classical circuit which contains only half of these recruitment reactions provides strong bistability. Thus these reactions can often be replaced; the attack on E1 or E2 can often be left to noise and, less frequently, is stimulated by the non-E nucleosomes. Interestingly, the creation of E1 or E2 sometimes occurs as a result of non-E nucleosomes ‘destroying’ themselves - recruiting enzymes that act on their own type. In contrast, E1 and E2 never recruit enzymes that destroy themselves, though they occasionally act to create the opposing E type.

Working circuits generally involve strong activity of the E1 and E2 nucleosomes, usually containing 4–6 reactions in which the E1 and E2 nucleosomes recruit enzymes that make modifications that move nucleosomes towards their own type, either creating themselves or attacking the opposing state (Fig. 3B; the consensus circuit contains 8 of these reactions). However, a reasonable number of circuits use only three reactions of this kind. In contrast, few circuits have E1 and E2 stimulating moves ‘away’ from their own type (Fig. 3C).

Fig. 3D shows that bistability requires at least 4 recruitment reactions in the circuit. However, the only working circuit with so few recruitment reactions is the classical circuit (Fig. 1C), while the consensus circuit (Fig. 2A) requires 8 such reactions. The minimal number of specific recruited enzymes required by the circuits (Fig. 3E) is substantially less than the number of recruitment reactions because in many cases a specific nucleosome type (e.g. 11) recruits enzymes that catalyse the same reaction (e.g. 0 to 1 at the first position) on two nucleosomes (e.g. 00 and 01). In these cases only a single enzyme is required; one that is sensitive to modifications at both positions on the recruiting nucleosome but is insensitive to the modification at the other position on its target nucleosome.

### Use of standard two-step cooperativity

Among the 202 motifs with balanced bistability, 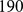 have at least one standard two-step positive feedback pathway, where E1 or E2 stimulate the reaction to create one of the intermediate (non-E) nucleosomes and also stimulate the reaction creating themselves from that intermediate nucleosome (Fig. 3F). Because this two-step pathway involves the successive action of TWO E1 (or E2) nucleosomes in their self-creation, it can produce a positive feedback with a dependence on the square of the number of E1 (or E2) nucleosomes, providing a more-than-linear response, or ultrasensitivity.

There are four possible standard two-step positive feedback pathways: two directions (towards E1 or E2) and two paths (over the R or non-R nucleosome). Circuits with one two-step pathway in each direction are most abundant but nearly as many circuits have just one such pathway (Fig. 3F). Involvement of the R nucleosome in these pathways is preferred, presumably because there are large numbers of these nucleosomes that need to be rapidly converted after replication.

We noticed that the standard two-step recruitment pathway nearly always includes an extra recruited reaction converting the intermediate type into the attacked E nucleosome type, pushing the intermediate “back”, against the flow of the two-step reactions. In fact, the standard two-step pathway with such a destabilized intermediate is seen in 185 of the 202 motifs that exhibit bistability (Fig. 3F).

**Figure 4:**
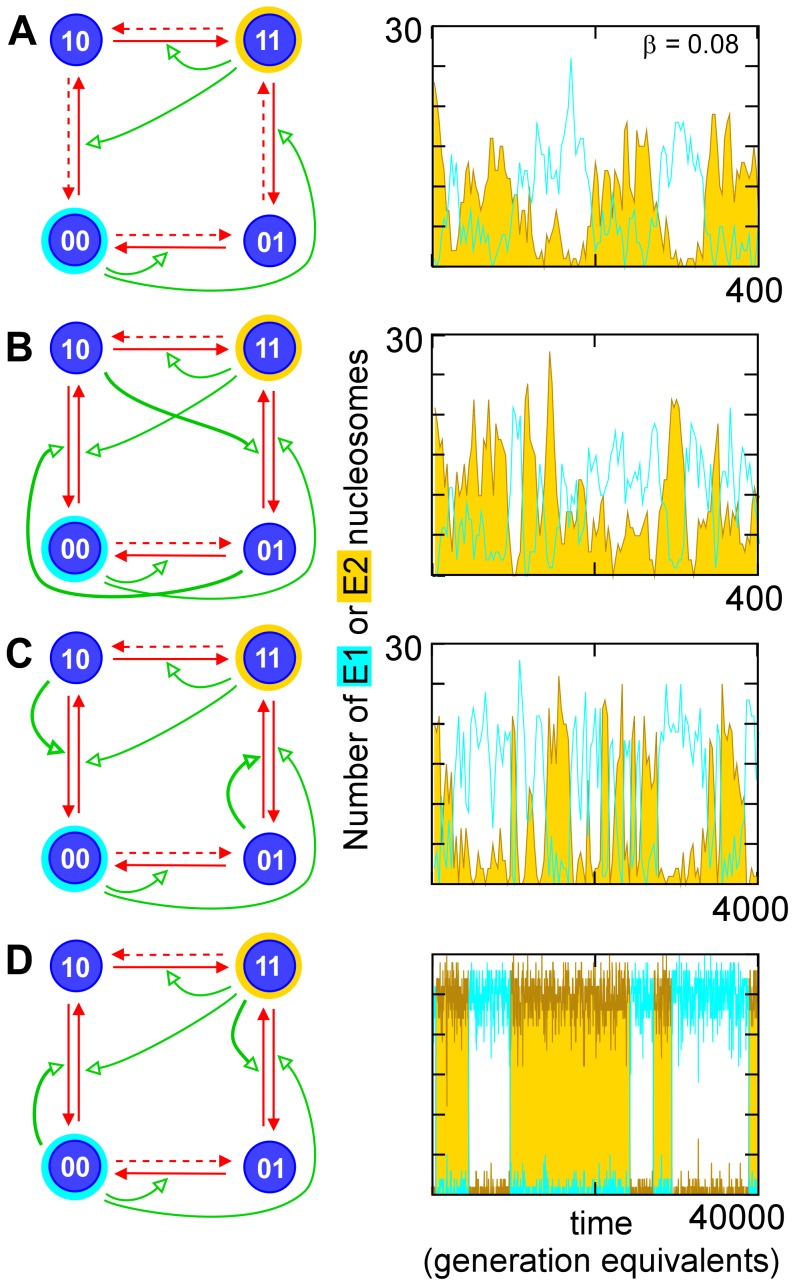
Requirement for destabilization of the intermediate for bistability by the standard 2-step motif. All circuits contain 2 standard two-step reaction pathways. The circuits in (B), (C) and (D) contain recruitment reactions in which the intermediate (non-E) nucleosomes are converted to the E nucleosome type that is attacked by the two-step pathway (thick green arrows). Catalysis of these reactions is stimulated by the other intermediate type (B), the intermediate itself (C) or the attacked E nucleosome type (D). Simulation was done without replication (each generation equivalent is 50 reaction attempts per nucleosome) and at high noise (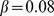). Note the different scales on the time axes.

This additional reaction is critical for bistability (Fig. 4). Simulations showed that a circuit with two standard two step pathways without intermediate destabilization gave poor bistability (Fig. 4A; in these simulations, replication was omitted and circuits were symmetrical in order to provide balance). Addition of destabilization by the intermediate converting itself to the opposing E type improved bistability somewhat (Fig. 4C). Strong bistability was obtained when the destabilization was catalyzed by the opposing E type (Fig. 4D), the type of reaction seen in the classical circuit and the 3 nucleosome-type system (Fig. 1).

This need for destabilization of the intermediate type can be understood as introducing a “loss” term for the intermediate nucleosome type that is necessary for ultrasensitivity. Considering the case where type 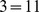 nucleosomes create themselves in two steps from type 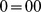 through type 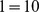 (see x02230x3 in Fig. 2):
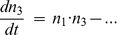
(2)

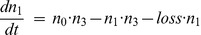
(3)giving a steady state occupation of the rare intermediate state 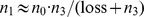. When this “loss” is sizeable, occupation of the intermediate state is sensitive to 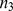 and eq. 2 then predicts ultra sensitive dependence of 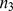 on itself. Notice that the “loss” term only supports ultra-sensitivity if the loss is not due to enzymes recruited by type 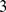 nucleosomes, as seen in the simulations in Fig. 4.

### Non-standard two-step cooperativity

**Figure 5:**
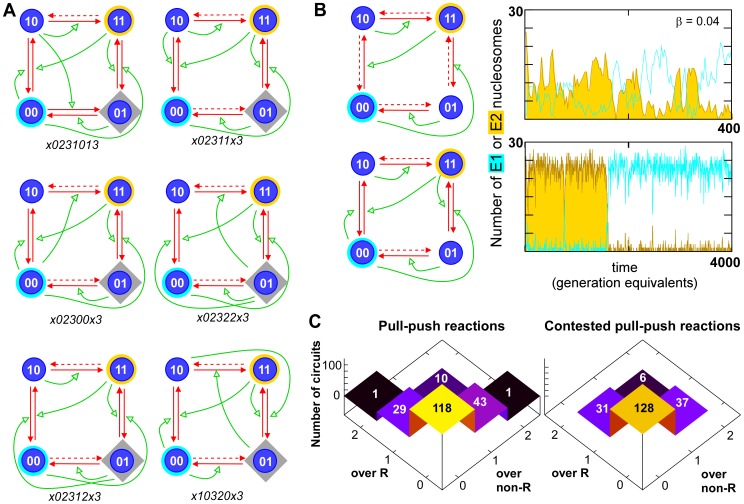
Bistable circuits without standard two-step cooperativity. (A) The exotic circuits. (B) Symmetrical circuits with two opposing pull-push motifs (upper) are not substantially bistable, while circuits with two destabilized pull-push motifs (lower) are. Simulations were without replication. (C) Left panel: Numbers of circuits among the 202 bistable E-opposite/R-out circuits with 0, 1 or 2 pull-push positive feedback pathways (left) or destabilized pull-push pathways towards either E1 or E2 shown according to whether the pathway is over the R-state, or the non-R state.

Surprisingly, 12 of the E-opposite/R-out circuits work without any of these standard two-step positive feedback pathways. This group of ‘exotic’ circuits is comprised of 6 unique circuits, each having a symmetrical twin (Fig. 5A).

Five of these circuits contain one or more reaction motifs that provide a novel form of two-step cooperativity (the exception is x10320x3). In these ‘pull-push’ reactions an E nucleosome recruits an enzyme that attacks the opposite E type, converting it to one of the intermediate types (R or non-R), and the intermediate recruits an enzyme that converts its own type to the E type (Fig. 5B). Because the E nucleosome acts to create the recruiter AND the target for the reaction that creates its own type, this pair of reactions provides a positive feedback that can exhibit an ultrasensitive dependence on the number of E nucleosomes, provided that the intermediate state is again destabilized. Simulation of a minimal circuit with two pull-push reactions (without replication) shows that this motif alone is not able to provide bistability (Fig. 5B). However, adding a loss of the intermediate, due to a self-creation reaction catalyzed by the attacked E type, provides robust bistability even at high noise levels.

**Figure 6:**
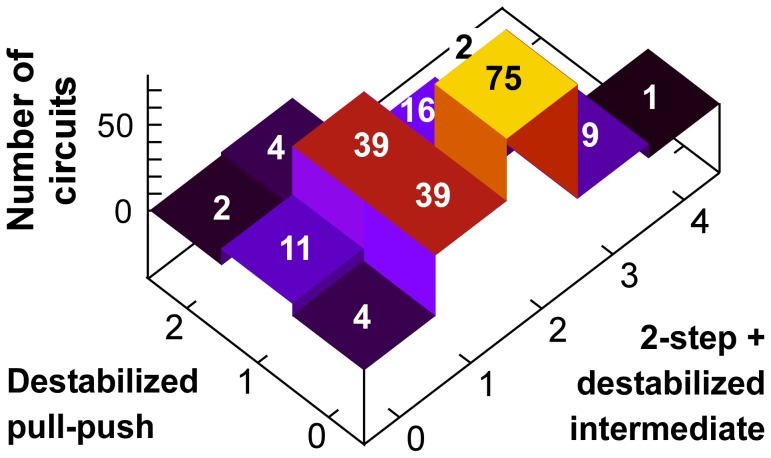
Combinations of the two cooperativity motifs used in the bistable circuits. The histogram shows the number of destabilized 2-step motifs and destabilized pull-push motifs used in the 202 bistable E-opposite/R-out circuits. Although a majority of circuits use only the 2-step motif, many circuits contain at least one of each motif.

This destabilized pull-push motif is present in 74 of the 84 bistable circuits that contain pull-push reactions (Fig. 5C). These reactions are therefore likely to play a role in strengthening the bistability in a large fraction of the 202 bistable E-opposite/R-out circuits. even when standard two-step cooperativity is present. Fig. 6 examines the frequencies of the destabilized pull-push and standard 2-step motifs in the 202 bistable E-opposite/R-out circuits. Only 4 circuits have neither of these motifs (2 symmetrical pairs), one being the x10320x3 circuit (Fig. 5A).

The x10320x3 circuit (Fig. 5A) and its symmetrical counterpart do not contain either a standard two-step motif or a pull-push motif. However recruitment from 10 act to support the 01 type that attacks 10, and thus recruitment reactions around 10 represent a variant destabilized pull-push motif. At the same time, the 01 type is occupied simultaneously with type 11, and together they provide a 2-step recruitment 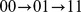. Thus the fact that this exotic motif is stable even up to noise level 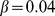 may well select that it integrates the two recruitment paths that both lead to ultrasensitivity.

### Expanded possibilities for epigenetic circuit evolution

**Figure 7:**
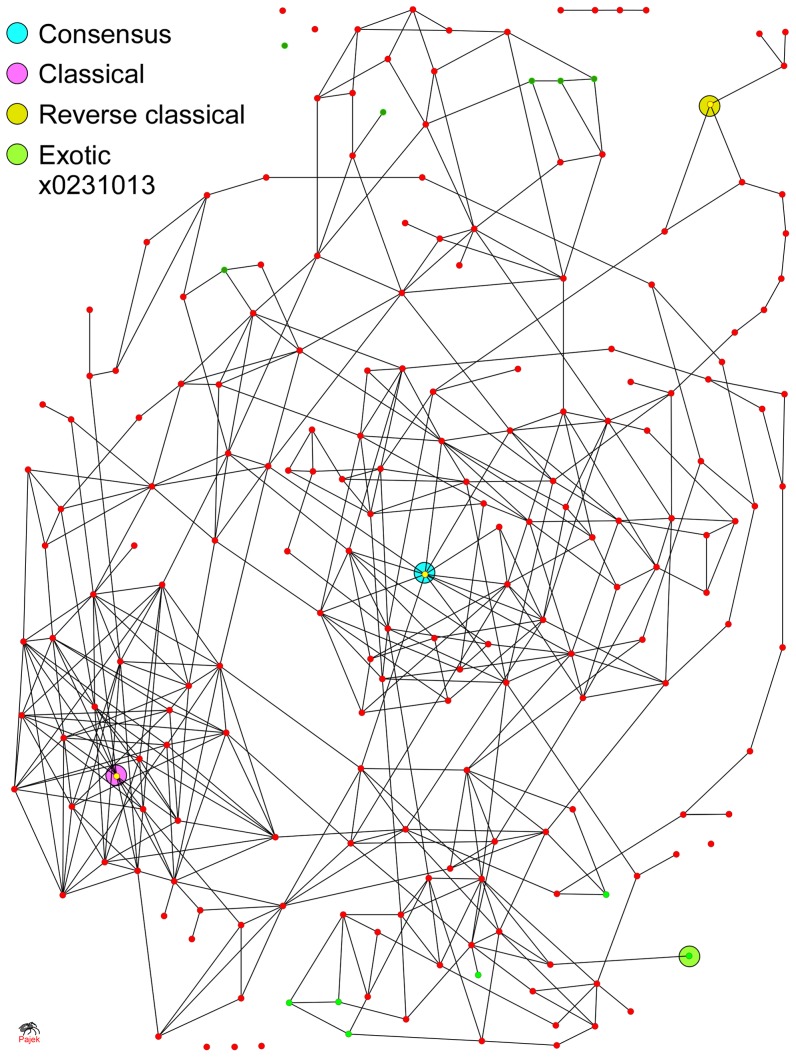
An evolutionary network of epigenetic circuits. The 202 working E-opposite/R-out circuits are shown as nodes, with links between nodes indicating a single reaction difference (i.e. different at only one digit of the circuit code). The green nodes are the 12 exotic circuits, lacking standard two-step cooperativity. Six of the exotic circuits are located near the bottom of the network; their 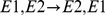 symmetric versions are located near the top. The locations of selected circuits are indicated.

The large number of different bistable circuits revealed in our screen suggests that different organisms, different cells and different genomic regions could utilize different variations of a 4-nucleosome-type modification system to achieve epigenetic regulation. To examine how such differences could arise by evolution, we looked at the ‘connectedness’ of the different circuits. Fig. 7 shows each of the 202 bistable circuits as nodes in a network, each linked to circuits that have only one reaction catalyzed differently.

The network tends to be clustered into two main groups, centered around the consensus circuit 00033033 and a more peripheral cluster centered around the classical circuit x003xx30 where the non-R paths are less catalyzed.

Remarkably, 191 of the circuits are connected into one network. This connectedness is surprising, given the strong limitations we put on our search space for circuit motifs, and means that the large variety of bistable circuits can be explored through a succession of relatively small evolutionary changes, without loss of bistability. Sequences of single enzymatic replacements can connect circuits that have no common recruitment, for example motif 0x2131x3 and x003xx3x shown explicitly in Fig. 7. Stepping through this network, a system may maintain basic epigenetic and regulatory properties while exploring subtle differences due to different combinations of recruitment processes. This evolutionary network resembles evolution of RNA sequences, which traverses far ranging but connected neutral plateaus of primary sequences which fold in identical secondary structures [26]. A degeneracy has also been suggested for regulatory networks that can digitalize morphogen gradients [27].

## Discussion

By sampling histone code circuits for their ability to support heritable bistability we have addressed the minimal requirements for obtaining epigenetics, defined as the ability to remember one of several possible states across several cell generations. Our findings are also important because histone code circuits with potential for bistability allow genetic regulation that can be ultrasensitive or graded, depending on the size of the system and the extent to which it couples to a noisy catalytic environment in the cell [21].

Our search was constrained to a “circuit code” space which was limited in both the number of considered modifications and in the scope for regulating each transition. In spite of these limitations, we found many solutions, including some unexpected new regulatory designs for bistable feedback systems. Thus, having two histone positions that can be modified AND having reading and writing enzymes that can distinguish the four resulting nucleosome types allows a large variety of reaction circuits that can generate stable and heritable alternative modification states.

This increased number of circuits results from the increased number of pathways available for indirect cooperativity, where positive feedback involves two successive recruitment reaction steps. This indirect cooperativity can be achieved by two kinds of two-step positive feedback. In the standard pathway [11], a nucleosome type recruits enzymes that catalyse a sequence of two steps:one step to create an intermediate nucleosome type that is different from it by one modification and a second step to create its own type. We discovered a new cooperativity motif, the pull-push reaction, in which the second step is clarified out by the intermediate type recruiting the enzyme that converts itself. To generate ultrasensitivity, both cooperativity motifs require that the intermediate nucleosome type is destabilized by a recruitment reaction that pushes it in the opposite direction.

Our analysis reveals a number of ‘rules’ for generation of such epigenetic circuits: 1) The circuit should contain at least one two-step intermediate-destabilized pathway of either the standard or pull-push type. There were only 2% exceptions to this rule among our accepted motifs. 2) It is easier to produce working circuits if the alternative dominant nucleosome types are different at both modification positions, that is, are separated by 2 reaction steps. This reflects the ease of producing two-step cooperativity. 3) It is easier to produce working circuits if the new nucleosomes inserted after replication are not one of the alternative dominant nucleosome types. This helps avoid biasing the system too heavily towards one dominant state. 4) The dominant nucleosome types are highly active in recruiting enzymes that create their own type or destroy the opposing dominant type, and never self-destruct, that is, recruit enzymes that change their own type. 5) Self-destruction is only seen for intermediate nucleosome types. These rules could be relaxed with removal of some of the restrictions we placed on the circuits. However, we believe that they are likely to be general features of nucleosome-based epigenetic systems.

Finally we found that conversion of one circuit into almost any other circuit can occur by a succession of small changes that retain heritable bistability, a feature that should facilitate circuit evolution. This plasticity is consequence of the fact that the two motifs that generate cooperativity are only one “mutation” away from each other in the sense that only one recruitment separates them from each other.

Although our restricted 4-nucleosome-type system is capable of surprisingly complex behavior, it is extremely simple compared to real systems. Nucleosomes are known to be modified in multiple ways at many positions, and modifying enzymes are likely to be sensitive to combinations of these modifications in complex ways. Thus, our analysis indicates that real systems have huge potential for generating multiple stable and heritable nucleosome modification states.

## References

[1] Taverna SD, Li H, Ruthenburg AJ, Allis CD, Patel DJ (2007) How chromatin-binding modules interpret histone modifications: lessons from professional pocket pickers. Nat Struct Mol Biol 14: 1025–1040. 17984965 10.1038/nsmb1338 PMC4691843

[2] Strahl BD, Allis CD (2000) The language of covalent histone modifications. Nature 403: 41–45. 10638745 10.1038/47412

[3] Wang Z, Zang C, Rosenfeld JA, Schones DE, Barski A, et al (2008) Combinatorial patterns of histone acetylations and methylations in the human genome. Nat Genet 40: 897–903. 18552846 10.1038/ng.154 PMC2769248

[4] Garcia BA, Pesavento JJ, Mizzen CA, Kelleher NL (2007) Pervasive combinatorial modification of histone H3 in human cells. Nat Methods 4: 487–489. 17529979 10.1038/nmeth1052

[5] Pesavento JJ, Bullock CR, LeDuc RD, Mizzen CA, Kelleher NL (2008) Combinatorial modification of human histone H4 quantitated by two-dimensional liquid chromatography coupled with top down mass spectrometry. J Biol Chem 283: 14927–14937. 18381279 10.1074/jbc.M709796200 PMC2397456

[6] Ruthenburg AJ, Li H, Patel DJ, Allis CD (2007) Multivalent engagement of chromatin modifica-tions by linked binding modules. Nat Rev Mol Cell Biol 8: 983–994. 18037899 10.1038/nrm2298 PMC4690530

[7] Ruthenburg AJ, Li H, Milne TA, Dewell S, McGinty RK, et al (2011) Recognition of a mononucleosomal histone modification pattern by BPTF via multivalent interactions. Cell 145: 692–706. 21596426 10.1016/j.cell.2011.03.053 PMC3135172

[8] Wang Z, Patel DJ (2011) Combinatorial readout of dual histone modifications by paired chromatin-associated modules. J Biol Chem 286: 18363–8. 21454653 10.1074/jbc.R111.219139 PMC3099652

[9] Musselman CA, Ramirez J, Sims JK, Mansfield RE, Oliver SS, et al (2012) Bivalent recognition of nucleosomes by the tandem PHD fingers of the CHD4 ATPase is required for CHD4-mediated repression. Proc Natl Acad Sci U S A 109: 787–792. 22215588 10.1073/pnas.1113655109 PMC3271909

[10] Braunstein M, Sobel RE, Allis CD, Turner BM, Broach JR (1996) Efficient transcriptional silencing in Saccharomyces cerevisiae requires a heterochromatin histone acetylation pattern. Mol Cell Biol 16: 4349–4356. 8754835 10.1128/mcb.16.8.4349 PMC231433

[11] Dodd IB, Micheelsen MA, Sneppen K, Thon G (2007) Theoretical analysis of epigenetic cell memory by nucleosome modification. Cell 129: 813–822. 17512413 10.1016/j.cell.2007.02.053

[12] Kaufman PD, Rando OJ (2010) Chromatin as a potential carrier of heritable information. Curr Opin Cell Biol 22: 284–290. 20299197 10.1016/j.ceb.2010.02.002 PMC3022377

[13] Moazed D (2011) Mechanisms for the inheritance of chromatin states. Cell 146: 510–518. 21854979 10.1016/j.cell.2011.07.013 PMC3244757

[14] Angel A, Song J, Dean C, Howard M (2011) A Polycomb-based switch underlying quantitative epigenetic memory. Nature 476: 105–108. 21785438 10.1038/nature10241

[15] Radman-Livaja M, Verzijlbergen KF, Weiner A, van Welsem T, Friedman N, et al (2011) Patterns and mechanisms of ancestral histone protein inheritance in budding yeast. PLoS Biol 9: e1001075. 21666805 10.1371/journal.pbio.1001075 PMC3110181

[16] Danzer JR, Wallrath LL (2004) Mechanisms of HP1-mediated gene silencing in Drosophila. Development 131: 3571–80. 15215206 10.1242/dev.01223

[17] Johnson A, Li G, Sikorski TW, Buratowski S, Woodcock CL, Moazed D (2009) Reconstitution of heterochromatin-dependent transcriptional gene silencing. Mol Cell 35: 769–81. 19782027 10.1016/j.molcel.2009.07.030 PMC2842978

[18] Margueron R, Justin N, Ohno K, Sharpe ML, Son J, et al (2009) Role of the polycomb protein EED in the propagation of repressive histone marks. Nature 461: 762–767. 19767730 10.1038/nature08398 PMC3772642

[19] Wysocka J, Swigut T, Milne TA, Dou Y, Zhang X, et al (2005) WDR5 associates with histone H3 methylated at K4 and is essential for H3 K4 methylation and vertebrate development. Cell 121: 859–872. 15960974 10.1016/j.cell.2005.03.036

[20] Sedighi M, Sengupta AM (2007) Epigenetic chromatin silencing: bistability and front propagation. Phys Biol 4: 246–255. 17991991 10.1088/1478-3975/4/4/002 PMC2267688

[21] Sneppen K, Micheelsen MA, Dodd IB (2008) Ultrasensitive gene regulation by positive feedback loops in nucleosome modification. Mol Syst Biol 4: 182. 18414483 10.1038/msb.2008.21 PMC2387233

[22] David-Rus D, Mukhopadhyay S, Lebowitz JL, Sengupta AM (2009) Inheritance of epigenetic chro-matin silencing. J Theor Biol 258: 112–20. 19174167 10.1016/j.jtbi.2008.12.021 PMC3034166

[23] Mukhopadhyay S, Nagaraj VH, Sengupta AM (2010) Locus dependence in epigenetic chromatin silencing. Biosystems 102: 49–54. 20655355 10.1016/j.biosystems.2010.07.012 PMC3676882

[24] Micheelsen MA, Mitarai N, Sneppen K, Dodd IB (2010) Theory for the stability and regulation of epigenetic landscapes. Phys Biol 7: 026010. 20526030 10.1088/1478-3975/7/2/026010

[25] Dodd IB, Sneppen K (2011) Barriers and silencers: A theoretical toolkit for control and contain-ment of nucleosome-based epigenetic states. J Mol Biol 414: 624–37. 22037584 10.1016/j.jmb.2011.10.027

[26] Schuster P, Fontana W, Stadler PF, Hofacker IL (1994) From sequences to shapes and back: a case study in RNA secondary structures. Proc Biol Sci 255: 279–284. 7517565 10.1098/rspb.1994.0040

[27] Cotterell J, Sharpe J (2010) An atlas of gene regulatory networks reveals multiple three-gene mechanisms for interpreting morphogen gradients. Mol Sys Biol 6: 425. 10.1038/msb.2010.74 PMC3010108 21045819

